# Family identification: a beneficial process for young adults who grow up in homes affected by parental intimate partner violence

**DOI:** 10.3389/fpsyg.2015.01249

**Published:** 2015-08-25

**Authors:** Catherine M. Naughton, Aisling T. O’Donnell, Orla T. Muldoon

**Affiliations:** Department of Psychology, Centre for Social Issues Research, University of Limerick, LimerickIreland

**Keywords:** parental intimate partner violence, psychological outcomes, family identification, social identity, psychosocial processes, mediation analysis, self-esteem, anxiety

## Abstract

Exposure to parental intimate partner violence (parental IPV) is a complex trauma. Research within social psychology establishes that identification with social groups impacts positively on how we appraise, respond to and recover from traumatic events. IPV is also a highly stigmatized social phenomenon and social isolation is a major factor for families affected by IPV, yet strong identification with the family group may act as a beneficial psychological resource to young people who grew up in homes affected by IPV. The current study, an online survey of 355 students (*M*_age_ = 20, 70% female), investigated if a psychosocial process, specifically identification with the family, may influence the relationship between the predictor, exposure to parental IPV, and outcomes, global self-esteem and state anxiety. Mediation analysis suggests that identification with the family has a positive influence on the relationship between exposure to parental IPV and psychological outcomes; exposure to parental IPV results in reduced family identification, but when family identification is strong it results in both reduced anxiety and increased self-esteem for young people. The findings highlight the importance of having a strong sense of belonging to the extended family for young people who were exposed to parental IPV, thus has implications for prevention, intervention, and social policy.

## Introduction

Recent work within the field of social identity, health, and wellbeing has established the benefits that subjective identification with a social group has on individuals’ wellbeing (Haslam et al., 2009). Group identification is a measure of one’s subjective internalization of a social group at both cognitive and affective psychological levels, where members obtain a sense of belonging and gain meaning from group membership. Moreover, strong identification with social groups not only affects the way we feel, think and behave, it also impacts on how we appraise and respond to stress (Gallagher et al., 2014).

Research by Sani et al. (2012) has established the protective role that identification with the family group has on wellbeing. Family identity may be described as an affiliative identity: an “invisible or background” identity which we are born into, and which we can be mobilized when required, for example in times of stress (Walsh et al., 2014). Affiliative identities provide a strong sense of belonging, which can function as a beneficial psychological resource in times of need. Despite the fact that families affected by parental IPV may be considered problematic (Bancroft et al., 2011), we propose that identification with that same family group may have an impact on the wellbeing of young adults who grew up in homes affected by parental IPV.

Intimate partner violence (IPV) is defined as a repeated pattern of coercive behaviors (physical and psychological) perpetrated by one partner over the other in an intimate relationship. IPV is a phenomenon which for the most part takes place within the home (Miller et al., 2015). Safe, stable and predictable family relationships are essential to child development (Unicef, 2006). However, Lieberman et al. (2011) suggest that children who grow up in homes affected by parental IPV are parented by “a parent who frightens and one who is frightened.” Concurring with this, a growing body of research over the last two decades has established that exposure to parental IPV has a negative impact on children’s cognitive, emotional, behavioral and social functioning (Holt, 2011) which can continue into adulthood (Artz et al., 2014). Based on the premise that it is mainly young children who are at greatest risk of being exposed to parental IPV (Fusco and Fantuzzo, 2009; Trocmé et al., 2013) much of the literature concentrates on young children (Hungerford et al., 2012). Where research does focus on young adults (aged 17–25) who have been exposed to parental IPV, there is a tendency for it to center on their own victimization/perpetration within intimate relationships (e.g., Rivera and Fincham, 2015).

Emergent literature has established an association between child exposure to parental IPV and young adults’ impaired wellbeing (Cater et al., 2015), but there is a dearth of research which explores the psychosocial factors which may influence this association. Exposure to parental IPV has been defined as a complex trauma as it may involve repeated exposure to aversive events from a very young age, with the perpetrator and victim (the child’s parents/caregivers) being known, loved and trusted by the child (Margolin and Vickerman, 2011). A large body of literature on recovery from trauma establishes the importance of various social factors in the link between experiencing aversive events and the development of resilience (Pynoos et al., 1999).

However, there may be inherent barriers to the availability of protective social factors for this population. Within the literature there is a strong link between IPV and social isolation (Levendosky, 2013). For children of families affected by parental IPV, social isolation may be derived from two interdependent factors. First, an established controlling tactic used by the perpetrator is to isolate their victims, including the children (Bancroft et al., 2011). Second, due to the stigma and shame associated with IPV, children/adolescents may self-exclude from social life (Buckley et al., 2007). Recent research suggests that young adults’ inability to disclose parental IPV throughout their childhood, despite an awareness of its existence, portrays an intrinsic level of isolation and secrecy among such young adults (Howell et al., 2014). This suggests that there may also be reduced opportunities for young adults who grew up in homes affected by parental IPV to become integrated within social groups outside the family, thus rendering their ties to their family all the more important.

Research more generally links family to positive psychological outcomes (see Elliott and Umberson, 2004). Initial findings also suggest that family may act as a beneficial psychological resource in the context of exposure to parental IPV across various age spans (children, adolescences, and young adults). For example, Owen et al. (2009) identified that child reports of family cohesion and relatedness to their primary attachment figure mediates the relationship between child reports of IPV and child adjustments (8–12 years). Similarly, Chanmugam (2014) found a strong sense of identity and belonging within mother–child–sibling relationships in a qualitative study of 12–14 year-old adolescents and their mothers from a refuge population. More recently, Miller et al. (2015) suggested that parental warmth may buffer the relationship between exposure to parental IPV and wellbeing in young adults. Therefore, despite the fact that parental IPV results in unpredictability and trauma within the home, it seems that children, adolescents, and young adults’ identification with their family or family members may influence their psychological outcomes. However, there is not yet conclusive evidence explicating the established variations in psychological outcomes (Kitzmann et al., 2003), nor the underlying processes which may influence the link between exposure to parental IPV and psychological outcomes. We therefore propose identification with family as a possible explanatory variable.

The current study used Edleson et al.’s (2008) measure of child exposure to domestic violence, hereafter referred to as *exposure to parental IPV*, which is in line with recent theoretical arguments to operationalize exposure to parental IPV broadly (Haselschwerdt, 2014). The exposure to parental IPV measure captures both physical and psychological violence, and as well as being validated with children (Edleson et al., 2008) it has also been validated as a measure of historical child exposure to parental IPV in young adults (Cater et al., 2015; Miller et al., 2015). To broaden our understanding of the impact of exposure to parental IPV on young adults, the current study focuses on young adults’ self-reports of both ongoing and historical exposure to parental IPV.

Further, to provide a more complete understanding of the impact of growing up in a home affected by parental IPV, outcomes within the present study were operationalized in terms of both short-term functioning (state anxiety) and long-term functioning (global self-esteem). Reviews and meta-analysis have clearly established associations between exposure to parental IPV and decreased self-esteem and increased anxiety in children (Evans et al., 2008; Holt et al., 2008; Haselschwerdt, 2014). Despite limited research with a young adult population, research has established associations between exposure to parental IPV and increased anxiety (Schiff et al., 2014; Miller et al., 2015) and reduced self-esteem (Davies et al., 2004) in young adults who grew up in homes affected by parental IPV. Global self-esteem, a measure of self-worth, develops over time, and aversive environments are deemed to have a negative impact on its formation (Rutter, 1993). State anxiety is a measure of in-the-moment or reactive anxiety. In the current study, participants completed the measure of exposure to parental IPV first, which acted to prime the participants and thus facilitated the capture of reactive anxiety.

Research within social psychology has established that identification with a social group can buffer the effects of trauma/stress, particularly for vulnerable groups (Haslam et al., 2009). As such, Branscombe et al. (1999) established that identification with one’s ethnic group was associated with increased self-esteem for minority group members, while Wakefield et al. (2013) demonstrated that support-group identification was linked to reduced anxiety in multiple sclerosis sufferers. However, the potential buffering effect of family identification has not been explored for young adults who grew up in homes affected by parental IPV.

Contextual factors such as gender and socioeconomic status (SES) may also impact on the relationship between exposure to parental IPV and young adults’ wellbeing. Cater et al. (2015) found gender by outcome interactions, with young women reporting significantly higher levels of anxiety than young men. However, as the young women also reported significantly higher levels of historical exposure to parental IPV than the young men, the authors cautioned about the presence of a gender reporting bias of both exposure to parental IPV and anxiety. There is also reason to believe SES may influence the impact of parental IPV on outcomes. The developmental literature suggests that it is the combination of childhood traumatic events together with an aversive environment that contributes to maladjustment (Herrenkohl and Herrenkohl, 2007; Gonzalez et al., 2014), and indeed in line with this, low SES is generally linked to poorer wellbeing (Lorant et al., 2003). In light of the previous findings on gender and SES, the current study also investigated the presence of differential effects for both gender and SES for exposure to parental IPV, family identification, anxiety, and self-esteem.

It is hypothesized that higher levels of reported exposure to parental IPV will predict higher levels of anxiety and lower levels of both family identification and self-esteem. Furthermore, based on social identity theory, family identification will mediate the association between exposure to parental IPV and both outcomes, anxiety and self-esteem.

## Materials and Methods

### Design

The current study was part of a larger cross-sectional online survey. Ethical approval was obtained from the Faculty’s Research Ethics Committee. Participants, from a predominately white university population, were invited to complete an online questionnaire. Participants gave their informed consent to partake in the study by ‘clicking’ a button. In acknowledgment of their time, participants were given the option to enter a prize draw for a €50 voucher. In line with the proposed mediational model, family identification (Doosje et al., 1995) was considered as a potential mediator in the association between exposure to parental IPV (Edleson et al., 2008) and both self-esteem (Rosenberg, 1965) and state anxiety (Marteau and Bekker, 1992).

### Participants

Since exposure to parental IPV is established as a pervasive problem (EU FRA, 2014), a convenience sample of university students was thought to provide sufficient variability within the regression model. While a total of 465 students completed the first measure, exposure to parental IPV, 23.66% failed to complete all measures, resulting in a final sample size of 355. There was no significant group difference [*t*(463) = -1.60, *p* = 0.11] in the level of reported parental IPV between participants who completed the entire survey (*n* = 355) and those who dropped out (*n* = 110). Of those who completed demographics, participants had a *M*_age_ = 20.07 years, SD_age_ = 2.08, 70.6% were female, and 46.5% were in receipt of income assessed government funding (suggesting that they are from low income backgrounds). 63% reported exposure to parental IPV (defined as a total score of exposure to IPV of 3 or greater), of those 36.1% stated that it was ongoing and 63.9% stated it was historical.

### Instruments

#### Demographics

As an indicator of SES, participants were asked to indicate if they were in receipt of income-assessed government funding to attend university. ‘Yes’ was coded as ‘lower SES’ and ‘No’ was coded as ‘higher SES,’ because funding is only provided to those with a sufficiently low income. Participants were also asked to provide their age and gender.

#### Predictor

##### Exposure to parental intimate partner violence

Edleson et al.’s (2008) validated scale for exposure to IPV was adapted to capture young adults’ self-reported exposure to parental IPV, both ongoing and historical, which was perpetrated by either or both of the participant’s parents/caregivers. To make the scale gender-neutral, the wording within each of the original items was altered so that references to ‘mother’ or ‘father’ were replaced by ‘parent/caregiver.’ For example, “How often did one *parent/caregiver* swear, yell or scream at, threaten the other *parent/caregiver* or call them names, fat, stupid or idiot etc.?” Participants rated the occurrence of both psychological and physical parental IPV on a 5-point Likert scale from 0 (never), 1 (rarely), 2 (sometimes), 3 (often), to 4 (a lot) (see supplementary material for individual items). To obtain maximum validity, 7 items (contact author for additional details) were totaled to give final scores between 0 and 28 for the exposure to parental IPV, with high scores indicating high exposure to parental IPV. Reliability was very good, with a Cronbach’s alpha of 0.88 for the current study.

##### Recency of exposure to parental IPV

Participants were asked to indicate the time frame of the most recent incident of exposure to parental IPV (ongoing, within 6 months, within 3 years, or over 3 years ago). This measure was collapsed to form a dichotomous variable, in that ‘ongoing’ refers to within 6 months, and ‘historical’ refers to more than 6 months ago.

#### Mediator

##### Family identification

Doosje et al.’s (1995) 4-item identification scale was used to assess family identification. Participants responded to items relating to their subjective, affective and shared identity within their family group by providing a rating from 1 (totally disagree) to 7 (totally agree). Means were calculated to give scores between 1 and 7, with higher scores indicating higher identification. Reliability was excellent, with a Cronbach’s alpha of 0.94 for the current study.

#### Outcomes

##### Anxiety

’sMarteau and Bekker (1992) 6-item scale was used to assess state anxiety. Participants responded to items relating to state anxiety from 0 (not at all) to 3 (very much so). Reliability was very good, with a Cronbach’s alpha of 0.85 for the current study. Items were totaled to give scores in the range of 0 to 18, with higher scores indicating higher anxiety.

##### Self-esteem

Rosenberg’s (1965) 10-item scale was used to assess self-esteem. Participants responded to items relating to global self-esteem from 1 (strongly agree) to 4 (strongly disagree). Reliability was very good, with a Cronbach’s alpha of 0.85 for the current study. The rated items’ mean was calculated to give a range of 1–4, with higher scores indicating higher self-esteem.

### Data Analysis Overview

The central aim of the current study was to investigate the impact of family identification on the link between exposure to parental IPV and both anxiety and self-esteem. Initial multivariate and follow-up univariate analyses of variance were performed to determine the need to control for any systematic group differences caused by SES and gender of the participant within the mediation model. Correlation analyses (Pearson’s *r*) were undertaken to identify associations between the variables of interest. Finally, to test the buffering effect of family identification, mediation analyses were performed.

Simple mediation models were analyzed using PROCESS model 4, which uses ordinary least squared regressions to yield unstandardized path coefficients for all pathways, as well as total, direct, and indirect effects (Hayes, 2013). Effects are deemed significant when the lower to upper limits of the accelerated 95% confidence intervals (CIs) do not pass through zero. The current analysis was undertaken both with and without bootstrapping. Bootstrapping involves drawing 1000 random samples from the data pool to estimate each pathway’s effects, with computed bias corrected and accelerated 95% CIs determining the significance of each pathway. Bootstrapping makes no assumptions about the normality in the sampling distribution and has superior control over type 1 errors when compared to non-bootstrapping (Preacher and Hayes, 2004).

## Results

### Group Differences

Results of the MANOVA identified a significant within subjects effect on exposure to parental IPV, family identification, anxiety, and self-esteem for both SES and gender of the participant. Follow-up univariate analysis of variance (ANOVA) testing the effect of gender of the participant on reported exposure to parental IPV, family identification, self-esteem, and anxiety proved non-significant. However, ANOVA testing the effects of SES revealed group differences of SES on exposure to parental IPV [*F*(1) = 6.46, *p* = 0.01] and family identification [*F*(1) = 6.81, *p* < 0.01]. As the exposure to parental IPV measure was composed of both ongoing and historical exposure to parental IPV, subsequent separate ANOVAs were also undertaken for ongoing exposure to parental IPV (*n* = 149), and also historical exposure to parental IPV (*n* = 264) to identify if differences between SES groups were present for exposure to both ongoing and historic parental IPV. For participants who reported ongoing exposure to parental IPV, ANOVA revealed group differences of SES for exposure to parental IPV [*F*(1) = 6.8, *p* = 0.01, $\eta_{p}^{2}$ = 0.05] and family identification [*F*(1) = 7.6, *p* < 0.01, $\eta_{p}^{2}$ = 0.06]. Specifically, within participants who reported ongoing exposure to parental IPV, those with lower SES reported significantly higher levels of exposure to parental IPV (*M* = 6.53, SD = 6.48) than those with higher SES (*M* = 4.05, SD = 4.04). In addition in this same group, those with higher SES reported significantly higher levels of family identification (*M* = 6.18, SD = 1.2) than those with lower SES (*M* = 5.44, SD = 1.69). These significant effects were maintained when we controlled for whether participants lived at home or had moved away. For participants who reported historical exposure to parental IPV, there were no significant SES group differences for either exposure to parental IPV or family identification. There were no significant differences between male and female participants for exposure to parental IPV, family identification, anxiety or self-esteem. Means and standard deviations of predictor, mediator, and outcome variables by gender and SES groups are presented in **Table 1** for exposure to ongoing parental IPV and in **Table 2** for exposure to historic parental IPV.

**Table 1:** Means (standard deviations) for exposure to parental intimate partner violence (IPV), family identification, self-esteem, and anxiety by socioeconomic status (SES) and gender of participant, for participants who reported IPV as ongoing.

	Male	Female	Higher SES	Lower SES
Exposure to parental IPV	5.57 (6.50)	4.74 (4.63)	4.05 (4.04)	6.53 (6.48)
Family identification	5.53 (1.52)	6.07 (1.37)	6.18 (1.20)	5.44 (1.69)
Self-esteem	2.83 (0.54)	2.70 (0.43)	2.75 (0.39)	2.71 (0.57)
Anxiety	5.70 (3.79)	6.65 (4.33)	5.71 (3.75)	7.53 (4.68)

**Table 2:** Means (standard deviations) for exposure to parental IPV, family identification, self-esteem, and anxiety by SES and gender of participant, for participants who reported IPV as historical.

	Male	Female	Higher SES	Lower SES
Exposure to parental IPV	5.41 (4.47)	5.24 (4.47)	5.19 (4.38)	5.44 (5.38)
Family identification	5.55 (1.76)	5.69 (1.56)	5.72 (1.56)	5.57 (1.69)
Self-esteem	2.79 (0.50)	2.71 (0.45)	2.73 (0.47)	2.74 (0.47)
Anxiety	7.06 (4.49)	7.79 (4.13)	7.79 (4.41)	7.33 (4.15)

### Inter-Correlations

Partial correlations (Pearson’s *r*) between variables are presented in **Table 3** (with SES as covariate), together with means, SD, and range. As predicted there was a moderate to large positive correlation between the predictor, exposure to parental IPV and the outcome, anxiety (*r* = 0.44) and a moderate to large negative correlation between the predictor, exposure to parental IPV, and the outcome, self-esteem (*r* = -0.39), and mediator, family identification (*r* = -0.50). There was a moderate to large positive association between family identification and self-esteem (*r* = 0.50) and a moderate to large negative association between family identification and anxiety (*r* = -0.54).

**Table 3:** Pearson correlation coefficients of exposure to parental IPV, family identification, self-esteem, and anxiety with SES as a covariate.

Variable	2	3	4	Mean (SD)	Range		*N*
					Min	Max	
(1) Exp. parental IPV	-0.50***	-0.39***	0.44***	4.54 (4.67)	0	25	465
(2) Family identification		0.50***	-0.54***	5.77 (1.55)	1	7	355
(3) Self-esteem	.		-0.61***	2.74 (0.47)	1.33	3.80	434
(4) Anxiety				7.08 (4.33)	0	18	431

Means, SD, and ranges are also included.

***p < 0.001, SD; Min, minimum; Max, maximum; N, sample size; Exp., exposure to.

### Mediation Analysis

**FIGURE 1:**
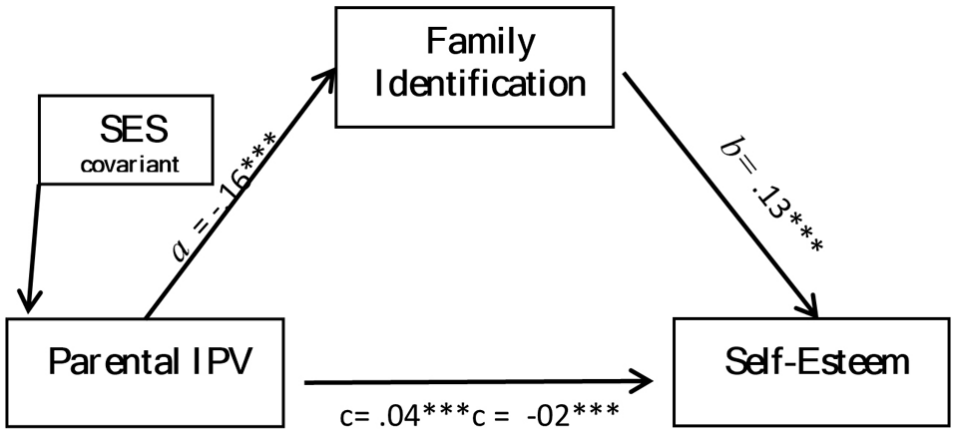
**Mediation of the effect of exposure to parental intimate partner violence (IPV) on anxiety by family identification.** ***P < 0.001.

**FIGURE 2:**
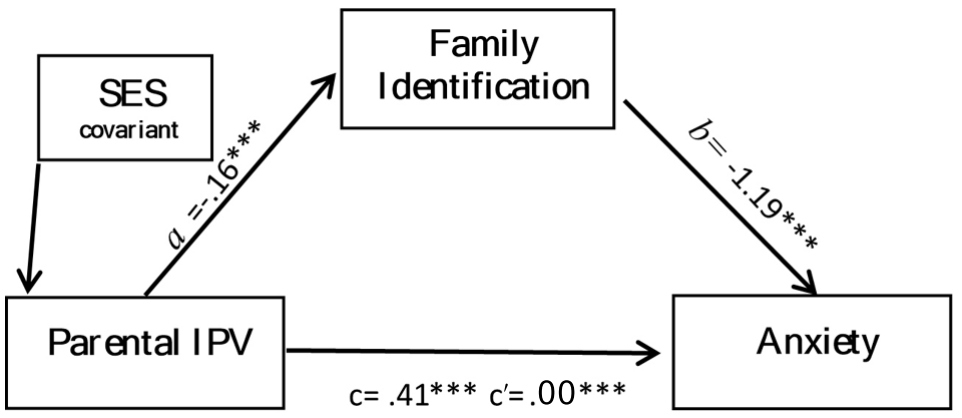
**Mediation of the effect of exposure to parental IPV on self-esteem by family identification.** ***P < 0.001.

Two simple mediation analyses including SES as a covariate were performed to analyze separately whether family identification influenced the association between exposure to parental IPV and both outcomes: anxiety (**Figure 1**) and self-esteem (**Figure 2**). Exposure to parental IPV predicted weaker family identification (*B* = -0.16, *p* < 0.001), which in turn predicted increased self-esteem (*B* = 0.13, *p* < 0.001) and reduced anxiety (*B* = -1.19, *p* < 0.001). There were significant indirect effects for both models (see **Table 4**); exposure to parental IPV affected both anxiety and self-esteem through family identification. Indirect effects, Model 1: exposure to parental IPV, family identification, anxiety [*B* = 0.19, 95%CL (0.14, 0.28)] and Model 2: exposure to parental IPV, family identification, self-esteem [*B* = -0.02, 95%CL (-0.03, -0.01)]. However, while significantly reduced, both direct effects remained significant; exposure to parental IPV was significantly associated with both anxiety (*B* = 0.22, *p* < 0.001) and self-esteem (*B* = -0.02, *p* < 0.001), while accounting for family identification. Furthermore in model 1, exposure to parental IPV and family identification collectively explained 33% of the variance in anxiety, while in model 2, exposure to parental IPV and family identification collectively explained 28% of the variance in self-esteem. Results were maintained when bootstrapping was employed. Non bootstrapping results are presented.

**Table 4:** Parameter estimates of the model examining the mediating role of family identification in the relationship between exposure to parental IPV and outcomes; anxiety and self-esteem.

			Path coefficients			
Outcome		Path	B	SE	95% CL	*R*^2^
Family identification	Parental IPV	*a*	-0.16***	0.02	(-0.19, -0.13)	
	**Model 1**					
Anxiety	Family ident	*b*	-1.19***	0.15	(-1.47, -0.90)	0.33
	Direct effect	c’	0.22***	0.05	(0.12, 0.31)	
	Indirect effect	ab	0.19	0.11	(0.14, 0.28)	
	**Model 2**					
Self-esteem	Family ident	*b*	0.13***	02	(0.09, 0.16)	0.28
	Direct effect	c’	-0.02***	0.01	(-0.03, -0.01)	
	Indirect effect	ab	-0.02	0.004	(-0.03, -0.01)	

Regression weights a,b,c, and c’ are illustrated in **Figures 1** and **2**. The 95% CI for a × b is obtained by the bias-corrected bootstrap with 1000 resamples. *Exposure to parental IPV* is the independent variables (*x*), Family identification is the mediator (*m*), and anxiety and self-esteem are outcome (*y*). *R*^*2*^ is the proportion of variance in *y* explained by *x* and *m*. CI (lower bound, upper bound) of 95% confidence interval (CIs). ***p < 0.001.

Note: In the initial ANOVA analysis we identified significant SES group differences for both exposure to parental IPV and family identification, for participants who reported exposure to parental IPV as ongoing. Therefore both mediation models were also undertaken with samples consisting of participants who reported only ongoing exposure to parental IPV (*n* = 118), then only historical exposure to parental IPV (*n* = 197). Significant indirect effects were maintained for both conditions; moreover these significant effects were also maintained when we controlled for whether participants live at home or had moved away.

## Discussion

The central aim of the current study was to investigate the role of family identification in the association between exposure to parental IPV and both anxiety and self-esteem. Mediation analysis identified that family identification buffered the association between exposure to parental IPV and both anxiety and self-esteem. There was a direct effect between exposure to parental IPV and both outcomes; high levels of exposure to parental IPV were associated with decreased levels of self-esteem and increased levels of anxiety. However there was also an indirect effect, in that family identification buffered the associations between exposure to parental IPV and psychological outcomes. Thus, despite the fact that higher levels of exposure to parental IPV were associated with weaker levels of family identification, participants who reported stronger levels of family identification also reported increased levels of self-esteem and decreased levels of anxiety. Family identification can therefore be said to play a positive role in the association between exposure to parental IPV and psychological outcomes for young adults. However, as higher levels of exposure to parental IPV were associated with weaker levels of family identification, those most affected are least likely to be able to draw on this beneficial resource.

This is the first study to consider family identification and belonging to the family group as an underlying psychosocial factor that may explain the association between exposure to parental IPV and psychological outcomes. As predicted, in line with previous research, stronger family identification was associated with more positive psychological outcomes (Sani et al., 2012). Moreover, strong family identification buffered the association between exposure to parental IPV and both anxiety and self-esteem. Current findings therefore build on previous research within the social identity tradition which document the explanatory role of family identification in the link between various traumas and psychological outcomes, for example: acquired brain injury (Walsh et al., 2014); multiple sclerosis (Wakefield et al., 2013); stroke (Haslam et al., 2008); and in the context of political violence in the Northern Ireland conflict (Muldoon et al., 2009) and Kosovo conflict (Kellezi et al., 2009). The current findings thus provide further evidence for the argument that social identities function as a ‘Social Cure’ (Jetten et al., 2012). The current findings also extends previous research which highlights the significance of affiliative identities – pre-existing groups which we are born into – in the context of trauma (Walsh et al., 2014). Additionally, they advance recent research within the literature on exposure to parental IPV, which investigates factors relating to family as potential mediators between child exposure to parental IPV and psychological outcomes across age spans. Both parental warmth (Miller et al., 2015) and family cohesion (Owen et al., 2009) are suggested as important mediators in that literature. The current study complements this research by including young adults’ perceptions of both ongoing and historical exposure to parental IPV.

Similar to the current study, the previous studies used a cross-sectional design with young Swedish adults (Miller et al., 2015) and African American children (Owen et al., 2009). In combination with these, our study provides compelling support for the importance of family to the psychological outcomes of young adults who grow up in homes affected by exposure to parental IPV. This may seem somewhat paradoxical, given that exposure to parental IPV may contribute directly to problematic family relationships and an aversive family environment. However, it should be noted that a strong integration within the family may be particularly important for this group. As discussed previously, due to the shame, stigma, and isolation associated with IPV, there are nuanced barriers to building strong links with social groups outside of the family for this at-risk population.

Contra to previous findings identified by Cater et al. (2015), who identified that Swedish young women reported significantly higher levels of historical child exposure to parental IPV than young men, the current study found no significant gender differences. In fact the trend was in the opposite direction, with young men reporting slightly (but not significantly) higher levels of exposure to parental IPV than young women. In the current study, young adults who reported ongoing exposure to parental IPV and who were from lower SES backgrounds reported significantly higher levels of exposure to parental IPV and significantly lower levels of family identification than their counterparts from higher SES backgrounds. However, there were no differences in the levels of either exposure to parental IPV and family identification based on SES for the participants who reported exposure to parental IPV as historical. One possible explanation for this is that those from lower SES backgrounds may live in smaller family homes therefore may be exposed to higher levels of ongoing parental IPV. Furthermore, as the current findings show, higher levels of exposure to parental IPV predict weaker levels of family identification, thus explaining the lower levels of family identification for participants from lower SES backgrounds who report ongoing exposure to parental IPV.

A strength of the current study was that two psychological measures of wellbeing were used; a global measure of self-esteem (long term functioning) and a reactive measure of state anxiety (short term functioning). This gives a more complete picture of the consequences of exposure to parental IPV for young adults. Global self-esteem develops over time, while state anxiety is a measure of “in the moment” anxiety levels. Completing the measure of exposure to parental IPV may be said to have primed the participants, and since the measure of state anxiety is reflective of current affective status, it therefore captures a more implicit and immediate response. As such, using both long term and reactive psychological outcomes, which have both been associated with resilience, allows for a more complete picture of the ongoing consequences of growing up in a home affected by parental IPV.

However, there are of course some limitations. For example, the current findings are not generalizable as participants in the current study could all be considered “high academic achievers” in that they attained sufficient grades to qualify for a university place, and this distinguishes them from the general population. A more pronounced effect may therefore have been identified in a high risk population, or indeed a general population sample.

Additionally, participants self-reported both ongoing and historical exposure to parental IPV; therefore the measure captures their awareness of the occurrence of parental IPV, but did not explicitly ask if they had directly witnessed the parental IPV. Furthermore, there are questions surrounding the validity of self-report measures of aversive childhood events within cross-sectional data (Widom et al., 2004). The accuracy of reporting exposure to parental IPV may in fact be a function of current psychological functioning. Individuals with low self-esteem, for example, may be motivated to attach meaning to their sub-optimal psychological functioning, therefore may be more likely to reflect on their childhood experiences in a more negative light (Horwitz et al., 2001). Furthermore, there may also be reciprocity between family identification and the reporting of exposure to parental IPV, as those with stronger identification may be less likely to report non-favorable family dynamics. However, it should be noted that participants’ family identification would not necessarily have been salient when completing the exposure to parental IPV measure, as this measure was completed prior to that of family identification.

Importantly, as longitudinal data is seen as a requirement to determine causality, the design of the current study (a cross-sectional study) is said to impede a conclusive causal interpretation. Although Hayes (2013) states that the limitations of data collection should not limit the statistical tools we use to help us understand the underlying processes which may be at play within our data, the findings must be said to be exploratory and not causal. The findings point to the need for longitudinal studies with at-risk children and young adults, which capture current measures of exposure to parental IPV, family identification and psychological outcomes at various time points. Future qualitative studies may also give an in-depth and nuanced understanding of the processes of family identification in the context of exposure to parental IPV. Furthermore previous research has established a co-occurrence of child exposure to parental IPV and direct child maltreatment (Herrenkohl et al., 2008). Future research is warranted which explores the buffering effect of family identification on cumulative trauma for young people who grew up in homes affected by parental IPV.

The current findings demonstrate the important insights which can be gleaned by a paradigm shift from individually focused research to research which explores the impact of psychosocial factors. As such, this study highlights the positive influence that strong identification with family can have on young people who grew up in homes affected by parental IPV. This beneficial effect was identified in both short and long term adaptation, and also for young adults who reported both ongoing and historical exposure to parental IPV. Participants, who reported high levels of exposure to parental IPV but also stronger family identification, may have mobilized this affiliative identity, which then may have functioned as a beneficial psychological resource to buffer their affective status in the face of stress. Furthermore, although the occurrence of parental IPV may contribute to a suboptimal family environment, the current findings suggest that identification with that same family may promote the development of positive self-esteem over time.

The first and paramount consideration when dealing with victims of IPV (including children) should be their physical and psychological safety. That said, given the secrecy that surrounds IPV, it is important that parents, the extended family and service providers are educated on the potential protective effects that a strong identification with family can have so that an inherent sense of belonging within the extended family can be promoted for young adults who grow up in homes affected by parental IPV.

## Conflict of Interest Statement

The authors declare that the research was conducted in the absence of any commercial or financial relationships that could be construed as a potential conflict of interest.
