# Supplementary material for: Family identification: a beneficial process for young adults who grow up in homes affected by parental intimate partner violence
Source: Front Psychol. 2015 Aug 25;6:1249. doi: 10.3389/fpsyg.2015.01249 (PMC4548083; doi:10.3389/fpsyg.2015.01249)
Supplement: Supplementary file 1 [file Data_Sheet_1.DOCX]

**Appendix**

**Exposure to parental IPV measure (adapted from Edleson et al., 2008)**

1. How often did one parent/step-parent swear, yell or scream at, threaten the other parent/step-parent or call them names (fat, stupid, idiot etc.)?
2. How often did one parent/step-parent stop the other, or made it difficult to do something they wanted to do, for example leave the house, go to the doctor, visit friends or relatives, use the phone or internet?
3. How often has a parent/step-parent broken or destroyed something on purpose for example punched a wall, threw something, smashed a picture?
4. How often has one parent/step-parent physically hurt the other, for example, hit, punched, kicked, choked, shoved, pulled their hair?
5. How often has one parent/step-parent threatened to use a knife, gun or other object to hurt the other?
6. How often has one parent/step-parent actually hurt the other with a knife, gun or other object?
7. How often did your parents/step-parents disagree, argue or fight?
